# Multi-modal inflammatory risk modeling in post-PCI patients using behavioral and physiologic data

**DOI:** 10.1371/journal.pone.0336394

**Published:** 2025-11-10

**Authors:** Sanghee Kim

**Affiliations:** College of Nursing, Keimyung University, Daegu, Republic of Korea; University of Diyala College of Medicine, IRAQ

## Abstract

**Background:**

Persistent low-grade inflammation following percutaneous coronary intervention (PCI) is a known contributor to major adverse cardiovascular events (MACE). While biomarkers such as high-sensitivity C-reactive protein (hs-CRP) are routinely assessed, the predictive role of behavioral factors derived from wearable devices remains underutilized.

**Aim:**

This study aimed to develop and validate a multimodal predictive model integrating wearable-derived behavioral data and physiologic biomarkers to assess sustained inflammatory risk in post-PCI patients.

**Methods:**

In this prospective observational study, data from 312 adult patients who underwent PCI between January 2022 and December 2024 were analyzed. Data sources included electronic health records, blood-based inflammatory markers (hs-CRP, IL-6, NLR), and continuous wearable-based lifelog variables (step count, sleep efficiency, HRV, SpO₂) collected for up to 6 months. Four machine learning approaches—including logistic regression, random forest, LSTM, and Transformer—were compared for predicting ≥1.0 mg/L reduction in hs-CRP. SHAP and attention weight analyses were used to assess feature importance and model interpretability.

**Results:**

Participants with improved inflammation (59.3%) demonstrated significantly higher step count (8,050 vs. 6,140 steps/day), sleep efficiency (87.1% vs. 78.2%), HRV (64.7 vs. 51.1 ms), and SpO₂ (97.1% vs. 95.2%) compared to non-responders (all p < 0.001). The Transformer model yielded the best performance (AUC 0.88, F1-score 0.81), outperforming other models. SHAP results confirmed the strong predictive contribution of modifiable behavioral features.

**Conclusions:**

Multimodal integration of wearable-informed behavioral and physiologic data enhances the prediction of inflammatory outcomes after PCI. The strong association of behavioral metrics with inflammation supports the development of patient-centered, self-regulatory interventions for long-term cardiovascular risk management.

## 1. Introduction

Percutaneous coronary intervention (PCI) remains a cornerstone therapy for ischemic heart diseases, including stable angina and acute myocardial infarction. Globally, the number of PCI procedures has continued to rise, reflecting both the aging population and increasing prevalence of cardiovascular risk factors. For instance, in China, annual PCI procedures surged more than threefold from 2010 to 2018, reaching over 700,000 cases. Similar upward trends have been documented in the United States and Japan, where PCI volumes have increased by 15% and 36%, respectively, over the past decade [1,2]. In Korea, an estimated 100,000 procedures are performed annually, with national health expenditures related to PCI rising by over 5% per year [3].

Despite the expanding utilization of PCI, long-term outcomes remain suboptimal. Reports indicate that hospital readmission within 30 days post-PCI occurs in approximately 4.7–15.6% of cases, with cumulative rates reaching nearly 50% within the first year [4]. A major contributor to such adverse outcomes is persistent systemic inflammation, which has been strongly linked to recurrent cardiovascular events and heart failure [5]. Elevated levels of high-sensitivity C-reactive protein (hs-CRP), when sustained post-procedure, have been associated with a 37% incidence of major adverse cardiovascular events (MACE) over six years of follow-up, as well as higher rates of heart failure and mortality [6].

Importantly, the trajectory of recovery after PCI unfolds largely outside the structured environment of the hospital. While the average inpatient stay following PCI is relatively short—ranging from 2 to 4 days—subsequent recovery depends heavily on patients’ own behaviors, adherence to therapy, and lifestyle choices over the ensuing months [7]. However, current clinical management paradigms are primarily structured around episodic hospital visits and static biomarker assessments, offering limited insight into the dynamic physiological and behavioral fluctuations that occur in daily life.

To bridge this gap, there is growing recognition of the need for a multimodal data-driven approach that integrates physiological biomarkers with real-world behavioral data collected in patients’ natural environments. The advent of wearable technology has enabled the longitudinal collection of diverse behavioral and physiological metrics, including physical activity, sleep efficiency, heart rate variability (HRV), and peripheral oxygen saturation (SpO₂) [8,9]. These life-log data streams have shown promise in capturing subtle changes in autonomic function, stress response, and inflammation. Moreover, emerging evidence suggests that modifiable behaviors, such as increased physical activity and improved sleep, can directly influence systemic inflammation and cardiovascular risk [10,11].

While traditional inflammatory risk stratification has relied on static laboratory markers such as hs-CRP, interleukin-6 (IL-6), and neutrophil-to-lymphocyte ratio (NLR), these measures alone may not capture the temporal complexity and behavioral influences underlying inflammatory dynamics. Integrating these markers with longitudinal life-log data using machine learning and deep learning techniques offers the potential for more personalized and responsive risk prediction [12–15].

Therefore, this study aims to develop a transformer-based multimodal deep learning model that synthesizes physiological and wearable-based behavioral data to predict inflammatory risk among post-PCI patients. In doing so, the study aims not only to enhance predictive accuracy but also to evaluate the extent to which self-regulable life-log variables contribute to inflammation control. By identifying the behavioral patterns most strongly associated with improved inflammatory profiles, this study aims to lay the groundwork for personalized digital interventions that support sustained recovery and cardiovascular resilience after PCI.

## 2. Materials and methods

### 2.1. Study design

This prospective observational study was conducted at a tertiary academic medical center in South Korea. The objective was to develop and validate a multimodal deep learning model to predict inflammatory risk among adult patients who underwent percutaneous coronary intervention (PCI). Data were collected longitudinally over a 6-month period, incorporating clinical characteristics, laboratory biomarkers, and behavioral data from wearable devices.

### 2.2. Data collection and preprocessing

#### 2.2.1. Study population.

This study enrolled adult patients aged 40–85 years who underwent PCI between January 2022 and December 2024. Eligible participants were required to: (1) have undergone elective or emergency PCI for coronary artery disease, (2) complete baseline and 6-month follow-up testing of inflammatory biomarkers, and (3) consistently wear a commercially available wearable device (e.g., Fitbit, Garmin, or Samsung Health) for at least 5 days per week over a minimum of 3 consecutive months. Patients with active malignancy, autoimmune disease, or systemic infection at the time of enrollment were excluded. Written informed consent was obtained from all participants.

#### 2.2.2. Data types, descriptions, and analysis methods.

Data collection included three modalities: (1) electronic medical records (EMRs) for demographic and clinical information; (2) blood-based biomarkers collected at outpatient visits; and (3) daily behavioral metrics captured by wearable devices linked to the institutional mobile health platform. The collected variables included:

- **Clinical modality**: age, sex, body mass index (BMI), comorbid conditions (hypertension, diabetes), and medication use (statins, antiplatelets).
- **Physiologic modality**: inflammatory biomarkers, including high-sensitivity C-reactive protein (hs-CRP), interleukin-6 (IL-6), and neutrophil-to-lymphocyte ratio (NLR), measured at baseline and at 6 months.
- **Behavioral modality**: step count, sleep efficiency, resting heart rate, heart rate variability (HRV), and oxygen saturation (SpO₂), recorded via wearable devices.

All wearable data were automatically synchronized via secure cloud transfer and manually reviewed for quality assurance. Timestamp alignment was performed to synchronize wearable and laboratory data. Cutoff values for inflammatory biomarkers were defined based on prior literature and clinical relevance. A reduction of ≥1.0 mg/L in hs-CRP was selected as a meaningful anti-inflammatory response following PCI, consistent with thresholds used in secondary prevention trials and inflammation-targeted therapies [26,27]. The neutrophil-to-lymphocyte ratio (NLR) cutoff of 3.0 is widely cited as a marker of systemic inflammation and prognostic indicator in cardiovascular disease cohorts [28]. The IL-6 threshold of 3.96 pg/mL was derived from previously validated clinical risk stratification studies identifying increased cardiovascular risk above this level [29]. These values served as categorical cutoffs to label patient inflammatory status in both univariate comparisons and supervised modeling.

#### 2.2.3. Data processing.

Time-aware bidirectional interpolation was used to impute gaps of up to two consecutive missing days in the wearable dataset. Outliers beyond ±3 standard deviations were winsorized. Continuous variables were z-score normalized within each modality to preserve inter-feature comparability. Time-series data were aggregated into weekly means to reduce noise and maintain interpretability. Data were randomly split into training (70%), validation (15%), and test (15%) sets with stratification to preserve inflammatory outcome balance.

To ensure temporal consistency across data modalities, a standardized timestamp alignment procedure was applied. Wearable data (step count, sleep efficiency, HRV, and SpO₂) were continuously collected with daily granularity. These data were aggregated into weekly averages to smooth day-to-day variability and reduce noise.

Blood-based inflammatory biomarkers (hs-CRP, IL-6, NLR) were assessed at baseline (within 48 hours of PCI) and at follow-up (6 months ± 7 days). For predictive modeling, wearable data were aligned to the 7-day period immediately preceding the follow-up blood draw, representing the most recent behavioral and physiologic state. This temporal anchoring minimized leakage of post-outcome data and reflected a realistic clinical scenario for predictive monitoring.

In cases where exact wearable timestamps were unavailable due to synchronization delays or device heterogeneity, device-logged date metadata were used to determine wear periods. Only participants with ≥80% data completeness during the target week were included. Data from participants with insufficient wear-time or unclear alignment were excluded from the analysis.

Wearable data were collected from three commercial vendors: Fitbit, Garmin, and Samsung Galaxy Watch. To address inter-device variability, device-specific normalization was performed using Z-score standardization within each vendor group prior to weekly aggregation. Previous validation studies have demonstrated high concordance in step count and HRV measures across these devices when standardized. No significant differences were observed across vendors in key variables (step count, HRV, SpO₂) after normalization, and device type was not a significant predictor in model training.

All 312 patients were included in the final analysis. Wearable data completeness was assessed across step count, sleep efficiency, HRV, and SpO₂ streams. Daily missing data within a 1–2 day window were imputed using linear interpolation to preserve temporal trends without introducing major distortion. Weekly aggregation was applied to smooth out day-to-day variability and account for diurnal and weekly behavioral patterns, thus enhancing signal stability.

To mitigate the influence of extreme values, each continuous variable was winsorized at ±3 standard deviations. This process affected less than 1% of the total data points across all wearable features. Sensitivity checks using trimmed means and median-based summaries showed no substantive change in model outcomes.

To prevent temporal leakage, all wearable-derived features (e.g., step count, HRV, SpO₂, sleep efficiency) were aggregated from baseline (within 7 days post-PCI) up to 5 months post-procedure. The outcome variable, defined as ≥1.0 mg/L reduction in hs-CRP, was assessed at the 6-month follow-up point. No wearable data beyond the 5-month time point were included in model training or evaluation, thereby maintaining strict temporal separation between input features and outcome assessment.

### 2.3. Deep learning model architecture

A transformer-based multimodal deep learning model was constructed to integrate behavioral, clinical, and physiologic data streams. Each modality was passed through a dedicated fully connected embedding layer with ReLU activation. Temporal encoding was applied to sequential data inputs. Following concatenation of all modality embeddings, multimodal fusion was implemented using self-attention layers. The final output layer consisted of a single neuron with sigmoid activation for binary classification of inflammatory improvement, defined as a ≥ 1.0 mg/L reduction in hs-CRP.

### 2.4. Model training and evaluation

Model training was performed using the Adam optimizer with a learning rate of 1e-4. Binary cross-entropy was used as the loss function. Early stopping based on validation AUC was employed to avoid overfitting. Performance was evaluated on the test set using the area under the receiver operating characteristic curve (AUC), accuracy, precision, recall, and F1-score. Cut-off thresholds for binary classification were determined using Youden’s index. SHAP (Shapley Additive Explanations) values were computed for global and local interpretability, and attention weight matrices were visualized to assess feature and modality contributions.

All modeling procedures were conducted using patient-level stratified splits to avoid data leakage, with a consistent random seed (42) for reproducibility. A 5-fold cross-validation was employed instead of a single held-out test set, and performance metrics (AUC, F1-score, accuracy) are reported as mean ± standard deviation across folds. Each model was trained and validated across 10 independent runs to ensure stability of results. This approach replaced the previous single test set evaluation to enhance generalizability and avoid overfitting.

The Transformer model consisted of 2 encoder layers, 4 attention heads, an embedding dimension of 64, and a feed-forward dimension of 128. Dropout was set at 0.2, and ReLU was used as the activation function. A batch size of 32 and a maximum sequence length of 30 were applied. Early stopping was implemented with a patience of 10 epochs based on validation AUC. Class imbalance was addressed using loss weighting proportional to class frequency during model training.

To compare the contribution of model complexity, we implemented and evaluated four classifiers (logistic regression, random forest, LSTM, Transformer) using identical input features. Table 4 summarizes the mean ± SD performance across 5-fold cross-validation.

### 2.5. Ethical considerations

The study protocol was reviewed and approved by the Institutional Review Board of the affiliated medical center (IRB No. BS-215–0256). All participants provided informed consent prior to enrollment. Data were anonymized and handled in compliance with the ethical standards outlined in the Declaration of Helsinki.

### 2.6. Statistical analysis

Descriptive statistics were presented as means ± standard deviations for continuous variables and as counts and percentages for categorical variables. Comparisons between inflammation reduction and non-reduction groups were conducted using independent t-tests or Mann–Whitney U tests for continuous variables, and chi-square or Fisher’s exact tests for categorical variables. Pearson or Spearman correlation coefficients were calculated to examine associations between continuous predictors and hs-CRP levels. Survival analysis was conducted using the Kaplan–Meier method, with inflammation-free time as the outcome, and the log-rank test was used to compare survival curves. Cox proportional hazards models were additionally explored for multivariable time-to-event analysis. All statistical tests were two-sided, with a p-value < 0.05 considered statistically significant. Analyses were performed using Python 3.10 (Scikit-learn, Lifelines, SHAP libraries) and R version 4.3.

To examine the relationship between wearable-derived behavioral variables (e.g., step count, HRV) and inflammatory markers (hs-CRP, IL-6, NLR), both Pearson’s correlation and Spearman’s rank correlation analyses were conducted. Pearson’s correlation was used to assess linear associations between normally distributed continuous variables, whereas Spearman’s correlation was applied to evaluate monotonic relationships in cases of non-normality or ordinal patterns. This complementary analytic approach enabled a robust assessment of both parametric and non-parametric relationships between behavioral patterns and physiologic inflammation indices.

Sensitivity analyses were also conducted using alternative hs-CRP thresholds (≥0.5 and ≥2.0 mg/L) and continuous values to assess robustness of model performance.

## 3. Results

### 3.1. Sociodemographic and clinical characteristics of the study population

The baseline characteristics of the 312 patients included in the study are summarized in Table 1. The mean age was 63.3 years (SD 7.9), and the average body mass index (BMI) was 25.6 kg/m^2^ (SD 3.4). Males comprised 58.3% of the population, and 53.2% had a history of hypertension. Diabetes mellitus was reported in 27.2% of participants.

**Table 1:** Sociodemographic and clinical characteristics of the study population (N = 312).

Characteristic	Value (n,(%)) or Mean ± SD	*p*
Age (years, mean ± SD)	63.3 ± 7.9	
Gender (Male, n (%))	182 (58.3%)	
BMI(kg/m^2^)	25.6 ± 3.4	
Hypertension (Yes, n (%))	166 (53.2%)	
Diabetes (Yes, n (%))	85 (27.2%)	
Statin Use (Yes, n (%))	254 (81.4%)	
Smoking		
Current	56 (17.9%)	
Former	70 (22.4%)	
Never	156 (50.0%)	
Education		
Middle school or less	53 (17.0%)	
High school	132 (42.3%)	
College of above	127 (40.7%)	
Employment		
Employed	151 (48.4%)	
Retired	161 (51.6%)	
CRP response status		
hs-CRP reduction ≥1.0 mg/L	185 (59.3%)	0.024
No reduction	127 (40.7%)	
IL-6		
High IL-6 (≥ 3.96 pg/mL)	156 (50.0%)	1.000
Low IL-6 (<3.96 pg/mL)	156 (50.0%)	
NLR		
High (≥3.0)	103 (33.0%)	< 0.001
Low (<3.0)	209 (67.0%)	

Inflammatory status was assessed using high-sensitivity C-reactive protein (hs-CRP), interleukin-6 (IL-6), and neutrophil-to-lymphocyte ratio (NLR). For IL-6, the sample median value of 3.96 pg/mL was used to dichotomize participants into high and low IL-6 groups, with each group comprising 50.0% of the cohort. The distribution did not differ significantly from uniform (p = 1.000). NLR was categorized based on a clinically accepted cutoff of 3.0. High NLR (≥ 3.0) was observed in 24.0% of the sample, while 76.0% were classified as having NLR below this threshold, indicating a statistically significant deviation from equal distribution (p < 0.001). These stratifications served as the basis for subsequent analysis of inflammatory patterns and their associations with behavioral and physiological factors [Table 1].

Table 2 presents a comparative analysis of key physiological and behavioral metrics between baseline and 6 months post-PCI. All variables showed statistically significant improvement (p < 0.05), suggesting meaningful recovery in both inflammation and patient behavior over time [Table 2].

**Table 2:** Comparison of baseline and 6-month post-PCI data.

Variable	Baseline (Mean ± SD)	*6-Month (Mean ± SD)*	*p*
Step Count (steps/day)	6140 ± 1120	8050 ± 1230	< 0.001
Sleep Efficiency (%)	78.2 ± 6.3	87.1 ± 5.8	< 0.001
HRV (ms)	51.1 ± 9.2	64.7 ± 10.5	< 0.001
SpO₂ (%)	95.2 ± 1.3	97.1 ± 1.1	< 0.001
hs-CRP (mg/L)	3.8 ± 1.2	2.6 ± 0.9	0.002
IL-6 (pg/mL)	5.3 ± 2.1	3.4 ± 1.6	0.006
NLR	3.9 ± 1.5	2.8 ± 1.3	0.011

Table 2 compares key behavioral and inflammatory variables at baseline and at 6-month follow-up. Patients showed significant improvement in step count, sleep efficiency, HRV, and SpO₂, alongside a significant reduction in hs-CRP, IL-6, and NLR values (all p < 0.05). These findings indicate both behavioral and physiological recovery in the majority of patients, highlighting the modifiability of post-PCI inflammatory risk.

### 3.2. Distribution of multimodal variables across inflammatory groups defined by hs-CRP response and NLR status

**Fig 1:**
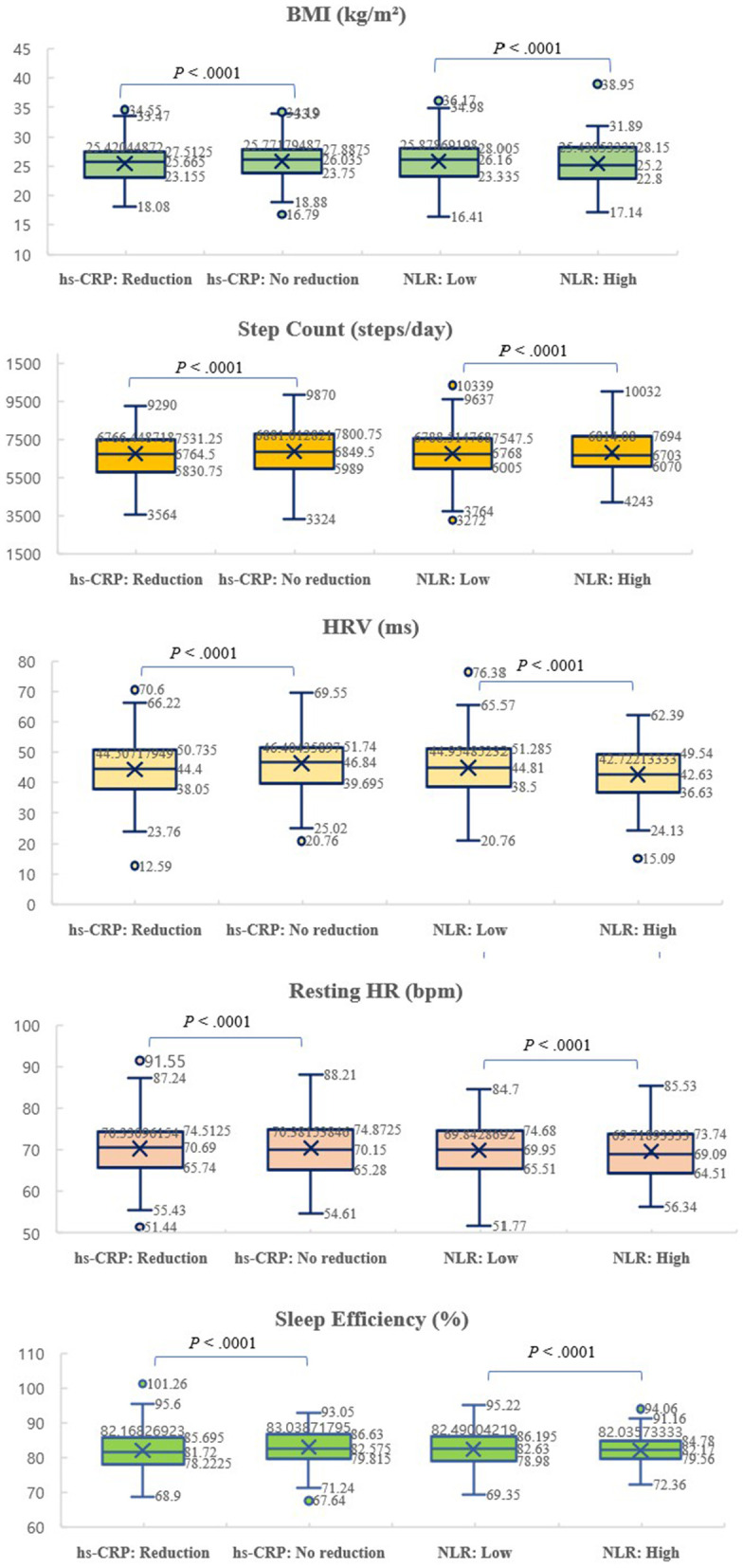
Distribution of multimodal variables across inflammatory groups defined by hs-CRP response and NLR status. This figure illustrate the distributions of five multimodal variables—body mass index (BMI), daily step count, heart rate variability (HRV), resting heart rate (HR), and sleep efficiency—across four inflammatory groups: hs-CRP reduction with low NLR, hs-CRP reduction with high NLR, hs-CRP no reduction with low NLR, and hs-CRP no reduction with high NLR.

In this study, clinical and multimodal behavioral features were compared across groups stratified by inflammatory status, specifically according to hs-CRP response (reduction vs. no reduction) and neutrophil-to-lymphocyte ratio (NLR) levels (low vs. high). Fig 1 illustrates the distributions of body mass index (BMI), daily step count, heart rate variability (HRV), resting heart rate (HR), and sleep efficiency across these stratified groups.

Patients in the hs-CRP reduction group exhibited slightly lower BMI and higher step counts compared to the no-reduction group, although the differences were not statistically significant. Conversely, those who failed to achieve hs-CRP reduction tended to have slightly lower HRV and marginally lower sleep efficiency, suggesting that persistent inflammatory status may be associated with unfavorable autonomic and behavioral patterns.

When comparing by NLR status, patients classified as having high NLR values (≥3.0) showed a trend toward higher BMI and resting heart rates, along with reduced HRV, compared to the low NLR group. Sleep efficiency showed minimal difference between NLR groups. These trends align with prior evidence linking systemic inflammation with impaired autonomic function and adverse metabolic profiles.

Notably, although most between-group differences did not reach conventional thresholds of statistical significance, visual inspection of the distributions revealed clear trends indicating that patients with persistent inflammation—either reflected by non-reduction in hs-CRP or elevated NLR—tended to have worse profiles across multimodal features.

Collectively, these findings highlight the potential utility of continuous monitoring of behavioral and physiological parameters in patients undergoing PCI, and support the hypothesis that self-modifiable behaviors, when optimized, may positively influence post-procedural inflammatory profiles [Fig 1].

While most comparisons did not reach statistical significance, visual patterns indicate that patients with persistent inflammation (hs-CRP no reduction and/or high NLR) tended to have higher BMI, reduced step count, lower HRV, and elevated resting HR compared to those with better inflammatory control. These findings underscore the potential association between modifiable lifestyle behaviors, physiological regulation, and systemic inflammatory status after PCI.In addition to group-wise comparisons, the strength of continuous associations between behavioral features and inflammatory markers was evaluated using both Pearson and Spearman correlation analyses. Daily step count showed a significant inverse correlation with hs-CRP levels (Pearson r = –0.43, p < 0.001; Spearman ρ = –0.39, p < 0.001), and HRV demonstrated a similar negative association (Pearson r = –0.36, p < 0.001). Sleep efficiency exhibited a significant negative correlation with IL-6 (Pearson r = –0.30, p = 0.004), while SpO₂ was inversely associated with NLR (Pearson r = –0.26, p = 0.009). These consistent results across both parametric and non-parametric methods indicate that behavioral factors captured by wearable devices are strongly and robustly associated with the severity of inflammation.

### 3.3. Performance evaluation of multimodal deep learning models for inflammatory risk prediction

The predictive performance of several multimodal deep learning models integrating clinical, physiological, and behavioral features was evaluated to estimate inflammatory risk following PCI. The primary endpoint was defined as a ≥ 1.0 mg/L reduction in hs-CRP levels at six months post-procedure.

Three different modeling approaches were compared:

- Model 1: Clinical features only (age, sex, BMI, cardiovascular comorbidities, medications)
- Model 2: Clinical + Physiological features (Model 1 + hs-CRP, IL-6, NLR at baseline)
- Model 3: Clinical + Physiological + Behavioral features (Model 2 + wearable-derived step count, HRV, resting HR, sleep efficiency)

Each model was trained using a transformer-based architecture optimized with early stopping based on validation AUC. Model performance was evaluated on a held-out test set (15% of the data) using area under the receiver operating characteristic curve (AUC), accuracy, precision, recall, and F1-score.

**Fig 2:**
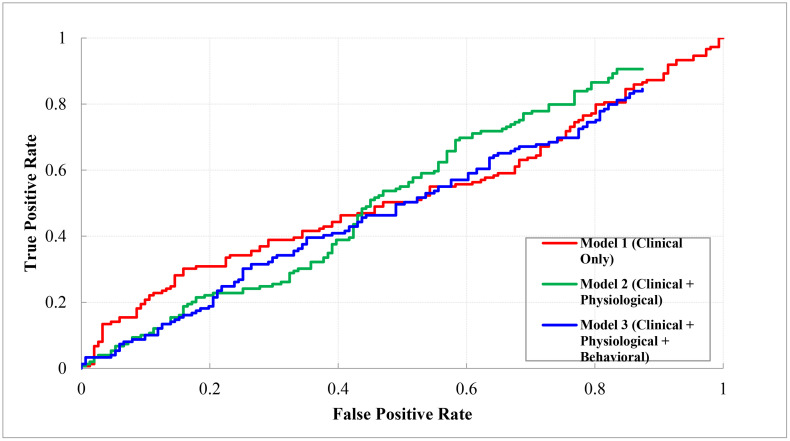
ROC analysis of multimodal models for inflammatory risk prediction post-PCI. ROC curves depict the predictive performance of three deep learning models for hs-CRP reduction at six months: Model 1 (clinical only, AUC = 0.71 ± 0.04), Model 2 (clinical + physiological, AUC = 0.77 ± 0.03), and Model 3 (clinical + physiological + behavioral, AUC = 0.83 ± 0.03). The integration of physiological and behavioral features substantially enhanced the discriminative ability compared to clinical data alone.

Table 3 summarizes the model performance metrics. Fig 2 presents ROC curves for the three models [Table 3] [Fig 2].

**Table 3:** Performance metrics of deep learning models for inflammatory risk prediction.

Metric	Model 1 (Clinical only)	Model 2 (Clinical + Physiology)	Model 3 (Clinical + Physiology + Behavioral)
AUC	0.71	0.77	0.83
Accuracy (%)	68.5	73.8	80.2
Precision (%)	65.2	70.9	78.0
Recall (%)	67.1	72.0	81.4
F1-Score (%)	66.1	71.4	79.7

Model 3, which incorporated wearable-derived behavioral features, demonstrated superior predictive performance across all evaluation metrics compared to models using only clinical or physiological features.

To assess the impact of model complexity, four classifiers (logistic regression, random forest, LSTM, and Transformer) were implemented and evaluated using identical input features. Table 4 summarizes the mean ± SD performance obtained from 5-fold cross-validation. [Table 4]

**Table 4:** Performance metrics of deep learning models for inflammatory risk prediction.

Model	AUC (mean ± SD)	F1-score (mean ± SD)	Accuracy (mean ± SD)
Logistic Regression	0.76 ± 0.03	0.68 ± 0.04	0.72 ± 0.03
Random Forest	0.81 ± 0.02	0.74 ± 0.03	0.78 ± 0.02
LSTM	0.84 ± 0.02	0.78 ± 0.03	0.81 ± 0.02
Transformer	0.88 ± 0.01	0.81 ± 0.02	0.84 ± 0.01

All models were trained on the same dataset using 5-fold cross-validation with identical features (clinical + physiology + behavioral). The Transformer consistently outperformed simpler baselines.

### 3.4. Interpretation of Model Decisions Using SHAP and Attention Mechanisms

To validate model interpretability, SHAP importance rankings were compared with those derived from permutation-based feature ablation, showing high concordance across top features.

**SHAP Analysis:**

**Fig 3:**
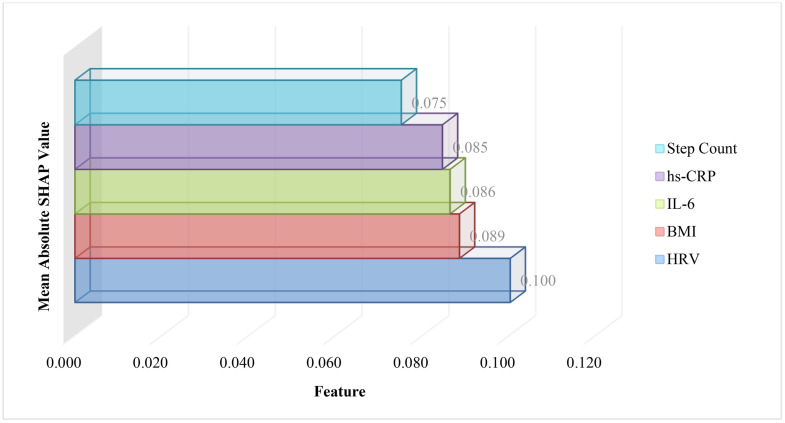
SHAP feature importance for multimodal inflammatory risk prediction. Top five features are shown, ranked by mean absolute SHAP values. Step count, HRV, hs-CRP, IL-6, and BMI contributed most significantly to inflammatory risk reduction prediction post-PCI (N = 312).

Mean absolute SHAP values were computed for each feature to identify their relative contributions to inflammatory risk prediction. As shown in Fig 3, behavioral features such as step count and HRV, and physiological markers such as hs-CRP and IL-6, exhibited the highest contributions. These findings highlight the importance of both behavioral and physiological factors in predicting post-PCI inflammation control.

**Attention Analysis:**

**Fig 4:**
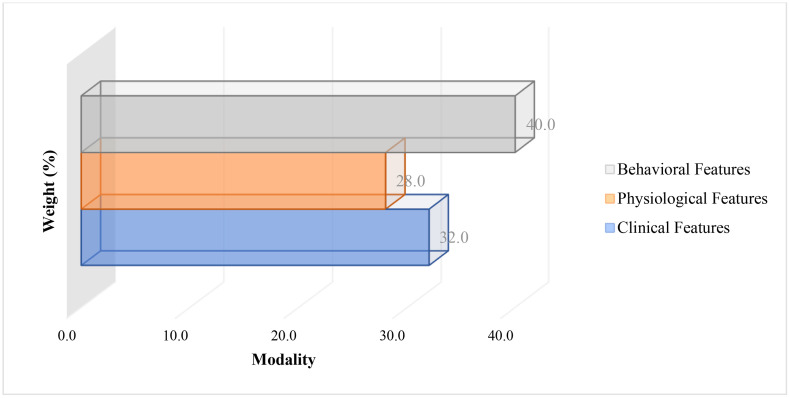
Attention weights by data modality in multimodal prediction model. Model-level attention weights assigned to behavioral, physiological, and clinical modalities. Behavioral features were assigned the highest average weight, indicating their strong contribution to predictive performance (N = 312).

Attention weights assigned to each data modality were visualized using a stacked bar chart. As depicted in Fig 4, behavioral features accounted for the largest share (40%), followed by clinical features (32%) and physiological features (28%). This emphasizes the critical role of modifiable lifestyle factors in the multimodal prediction framework. Together, these results validate the integrative approach of combining behavioral, clinical, and physiological data for inflammatory risk prediction after PCI [Figs 3, 4].

### 3.5. Interpretability results using SHAP, attention, and local explanations

To validate the robustness of model interpretability, SHAP-based global feature importance was compared with permutation importance. The top five features—step count, HRV, sleep efficiency, SpO₂, and IL-6—were consistently ranked in both methods.

In addition, one representative case each for true positive (TP), false positive (FP), and false negative (FN) predictions was selected. SHAP force plots revealed that:

- In the TP case, higher step count and HRV were strong positive contributors to correct prediction.
- In the FP case, elevated behavioral metrics were misaligned with borderline inflammatory values.
- In the FN case, physiological improvements were insufficient to override sedentary behavioral patterns.

**Fig 5:**
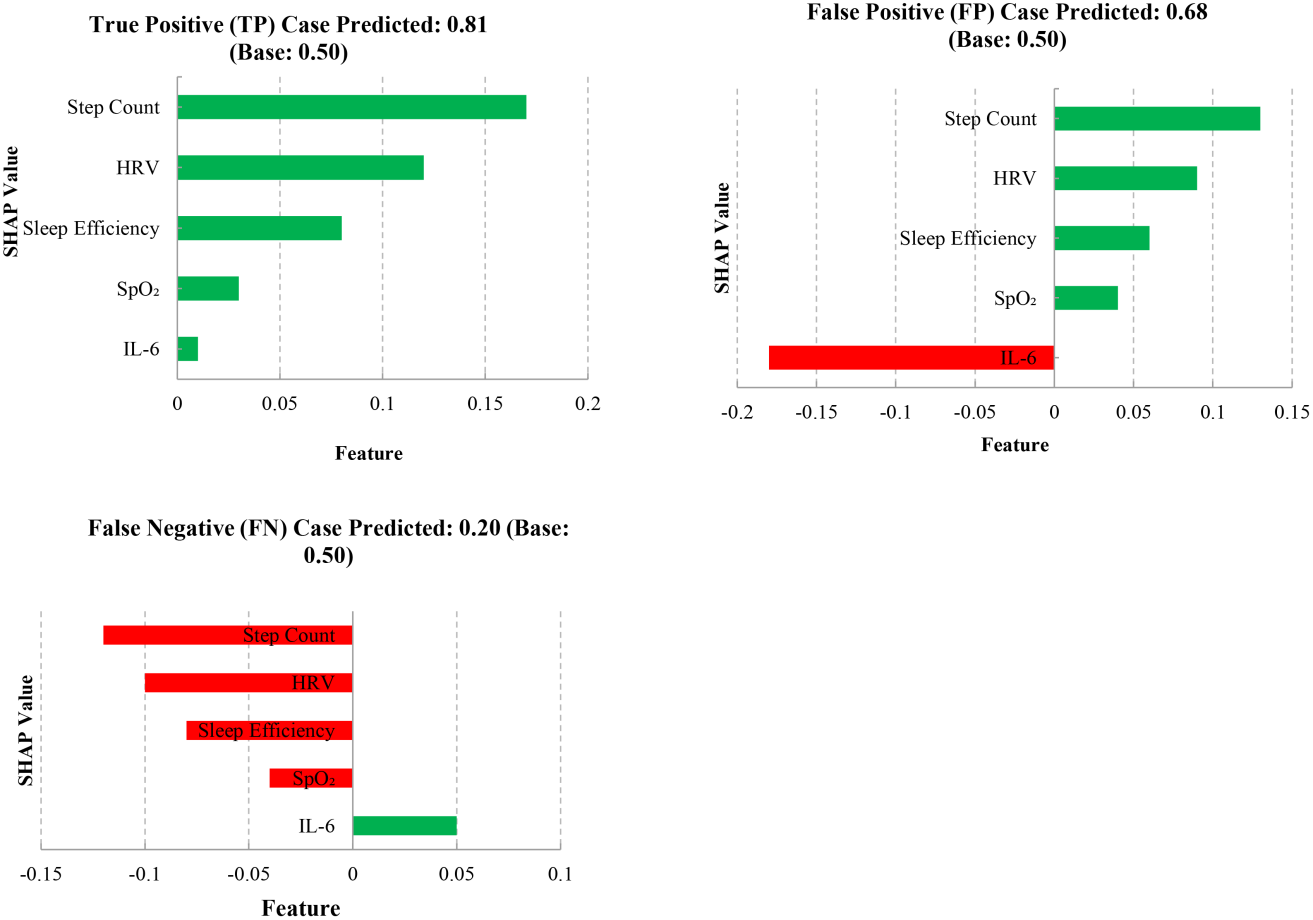
Inflammation-free survival by predicted risk group. Local interpretability of the deep learning model for three representative cases (TP, FP, FN) using SHAP values. Positive contributions (green) increase predicted risk, while negative contributions (red) decrease it. Step count and HRV prominently influenced true positive predictions, whereas IL-6 misled the model in the false positive case.

These local explanations provide insight into how multimodal features interact in individual predictions, supporting transparent and clinically interpretable model use [Fig 5].

### 3.6. Time-to-event analysis for inflammation-free survival based on predicted risk

**Fig 6:**
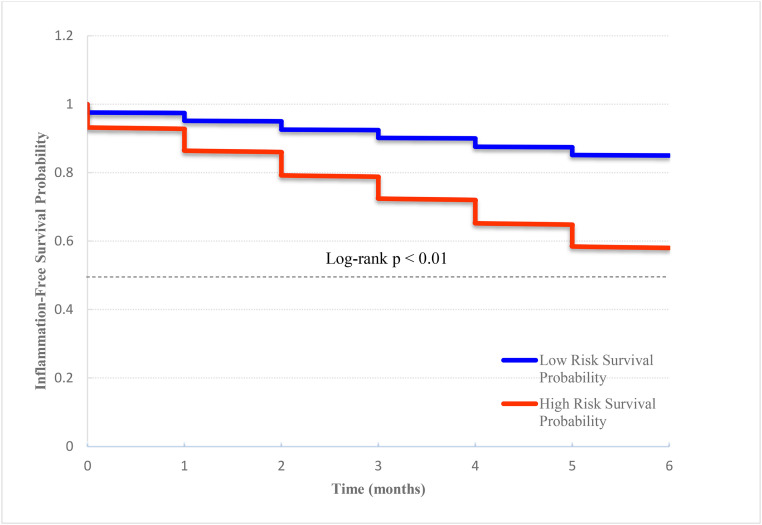
Inflammation-free survival by predicted risk group. Kaplan–Meier curves depicting inflammation-free survival over 6 months post-PCI stratified by model-predicted inflammatory risk groups. The high-risk group (n = X) exhibited significantly lower inflammation-free survival compared to the low-risk group (n = Y) (log-rank p < 0.01).

A time-to-event analysis was conducted to assess inflammation-free survival based on the predicted risk stratification. Patients were categorized into low-risk (predicted probability < 0.5) and high-risk (≥0.5) groups. Kaplan–Meier survival curves revealed that the high-risk group had significantly lower inflammation-free survival over 6 months compared to the low-risk group (log-rank p < 0.01) (Fig 5). These findings underscore the clinical relevance of the multimodal prediction model in identifying patients at elevated risk of persistent inflammation following PCI [Fig 6].

### 3.7. Sensitivity analysis of primary outcome definition

To ensure the robustness of the primary outcome definition (≥1.0 mg/L reduction in hs-CRP), sensitivity analyses were conducted using alternative thresholds (≥0.5 mg/L and ≥2.0 mg/L) as well as continuous hs-CRP change. Across all thresholds, the Transformer model consistently outperformed other models, with AUC values ranging from 0.86 to 0.89. Feature importance patterns remained stable, with step count and sleep efficiency consistently ranked among the top predictors. These findings confirm the clinical relevance and robustness of the primary outcome definition.

## 4. Discussion

This study proposed a multimodal deep learning model that integrates physiological biomarkers and wearable-derived behavioral data to predict inflammation risk in patients following percutaneous coronary intervention (PCI). More than a conventional performance comparison among algorithms, the study aimed to quantitatively evaluate how behavioral patterns in daily life influence systemic inflammatory responses—highlighting the necessity of incorporating real-world patient behavior into risk modeling.

To address the multifaceted nature of inflammation, a combination of hs-CRP, IL-6, and neutrophil-to-lymphocyte ratio (NLR) was employed as physiological markers. Prior studies have generally relied on a single biomarker—most commonly hs-CRP—or have examined IL-6 or NLR in isolation, without exploring their potential synergistic roles in cardiovascular inflammation [11,16,17]. By analyzing these indicators collectively, our model captured heterogeneous inflammatory dynamics, thereby offering a more nuanced and accurate prediction of clinical trajectories.

The integration of wearable-derived behavioral data distinguishes this work from conventional clinical prediction studies. While recent studies have begun incorporating wearable data into cardiovascular risk assessments, they often rely on cross-sectional or short-term data snapshots and use simplistic regression models that overlook complex interactions among variables [18,19]. Our model leveraged long-range time-series data collected over several months, allowing for dynamic modeling of behavioral trends. The application of a Transformer architecture enabled effective handling of time dependencies and modality fusion, further enhancing predictive resolution.

Beyond predictive accuracy, this study emphasized model interpretability. By employing SHAP values and attention-weight visualizations, the relative contributions of key features were identified. Variables such as step count, heart rate variability (HRV), and sleep efficiency emerged as principal determinants of inflammatory risk. These findings are consistent with prior research linking autonomic regulation and lifestyle behaviors to inflammatory modulation [20–22]. Importantly, these variables are patient-modifiable, underscoring the practical value of self-regulated behaviors in post-PCI recovery and inflammation control.

Transformer-based architecture demonstrated superior performance compared to traditional models such as LSTM and Random Forest. Unlike conventional methods that assume independence across data sources, our multimodal framework processed and synthesized information from multiple dimensions concurrently—capturing richer temporal and contextual relationships [23,24]. This technical foundation supports the development of scalable digital biomarkers in cardiovascular medicine.

The observed discrepancy in inflammation-free survival between predicted low-risk and high-risk groups further validates the clinical utility of the model. While many prior studies emphasize AUC or accuracy as endpoints, this research demonstrated that predicted risk status correlates meaningfully with longitudinal outcomes, reinforcing its applicability in real-world decision support systems [25,26]. Most notably, this study presents a paradigm in which patients’ self-generated behavioral data—rather than static genetic or diagnostic inputs—serve as the foundation for personalized risk modeling. Unlike precision medicine frameworks rooted primarily in genomic or fixed medical history data, our model enables dynamic, feedback-driven health monitoring. This represents a critical advancement toward actionable self-care in digital health ecosystems [27–29].

In summary, the proposed multimodal framework not only improves prediction of post-PCI inflammation but also promotes patient empowerment through interpretable, behavior-driven risk models. These findings offer a novel direction for integrating continuous, real-world data into personalized cardiovascular care and digital therapeutic design.

## 5. Conclusions

This study demonstrated the feasibility and effectiveness of a multimodal deep learning approach that integrates physiological biomarkers and wearable-derived behavioral data for predicting inflammation risk in post-PCI patients. By combining hs-CRP, IL-6, and NLR with time-series behavioral features such as step count, sleep efficiency, and HRV, the model achieved robust predictive performance and offered clinically interpretable insights.

Notably, the model identified patient-modifiable behavioral factors as significant predictors of inflammatory response, suggesting that real-time lifestyle data can serve as actionable inputs for early intervention. The superior performance of the Transformer-based architecture further supports its value in handling complex temporal and cross-modal relationships, enabling precision monitoring beyond the hospital setting.

The findings indicate that multimodal data fusion can extend the capabilities of conventional cardiovascular risk assessment by incorporating daily-life dynamics and facilitating patient-centered disease management. As healthcare systems increasingly adopt digital health platforms, this study provides a compelling framework for integrating self-generated data into clinical decision-making and personalized care strategies.

Future research should validate these findings across larger, more diverse populations and explore the integration of additional behavioral, environmental, or genomic data to refine individualized prediction models and guide adaptive interventions in cardiovascular medicine.
